# Rare Case of Valve-Sparing Root Reimplantation in a Patient With an Anomalous Circumflex Artery

**DOI:** 10.1016/j.jaccas.2024.102563

**Published:** 2024-11-27

**Authors:** Veronica Lorenz, Jama Jahanyar, Gebrine El Khoury, Laurent De Kerchove

**Affiliations:** Cardiovascular and Thoracic Department, University Hospital Saint-Luc, Brussels, Belgium

**Keywords:** aberrant coronary artery, anomalous left circumflex, bicuspid aortic valve, valve sparing

## Abstract

We present a case of valve-sparing root reimplantation in a patient with severe aortic regurgitation, in the setting of a bicuspid aortic valve with an aberrant circumflex coronary artery arising from the right coronary artery, thus rendering standard dissection of the aortic root during a David procedure challenging and risky.

## Graphical Abstract

**Figure:**
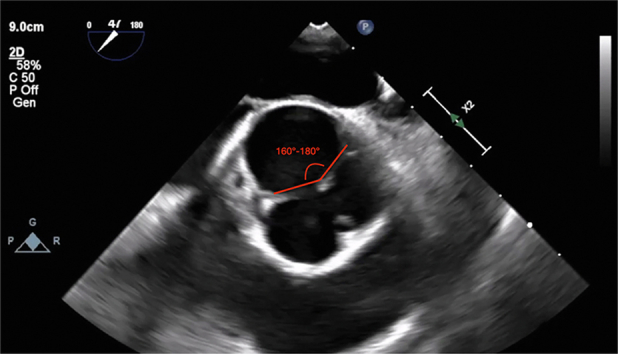

## History of Present Illness

The patient was a 30-year-old man, referred to our institution (University Hospital Saint-Luc, Brussels, Belgium) for a severely regurgitant bicuspid aortic valve (BAV). He was asymptomatic and had been followed up by the referring cardiologist for many years for an aortic murmur. Preoperative physical examination demonstrated normal vital signs and an early diastolic decrescendo murmur, best heard at Erb’s point.

**Take-Home Messages**

• BAVs can be associated with anomalous coronary arteries.
• Patients with BAVs are potential candidates for aortic valve repair, and appropriate preoperative imaging is important to demonstrate the relationship between the aortic root and the aberrant coronary artery.
• Mobilization of the ACA from the aortic annulus is key to performing a safe aortic valve or root procedure.

## Past Medical History

The patient had otherwise no significant past medical or surgical history and was in his usual state of health.

## Differential Diagnosis

The differential diagnosis of diastolic heart murmur includes early diastolic aortic regurgitation (AR), pulmonary regurgitation; mid- to late diastolic mitral stenosis, and Austin Flint murmur.

## Investigations

**Figure 1:**
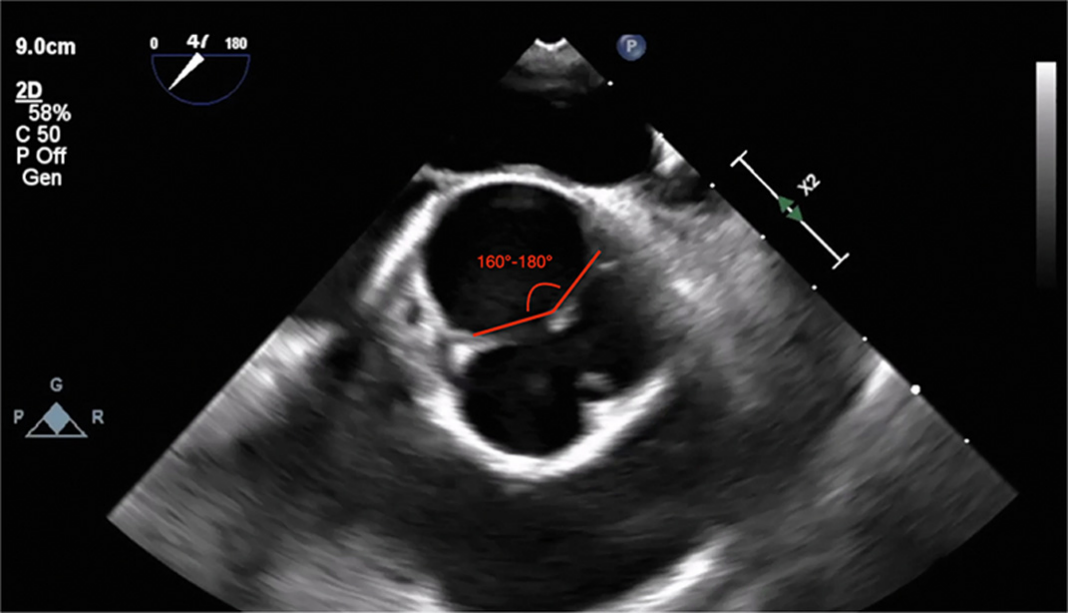
Midesophageal Aortic Valve Short-Axis View Type A bicuspid aortic valve symmetrical phenotype with a 160° to 180°commissural orientation. The commissural orientation is drawn and measured on a short-axis view and is defined as the angle measured on the nonfused cusp side between 2 lines joining each commissure to the central axis of the valve in diastole. C = compensation; G = gain; Gen = general; P = penetration; 2D = 2-dimensional.

**Figure 2:**
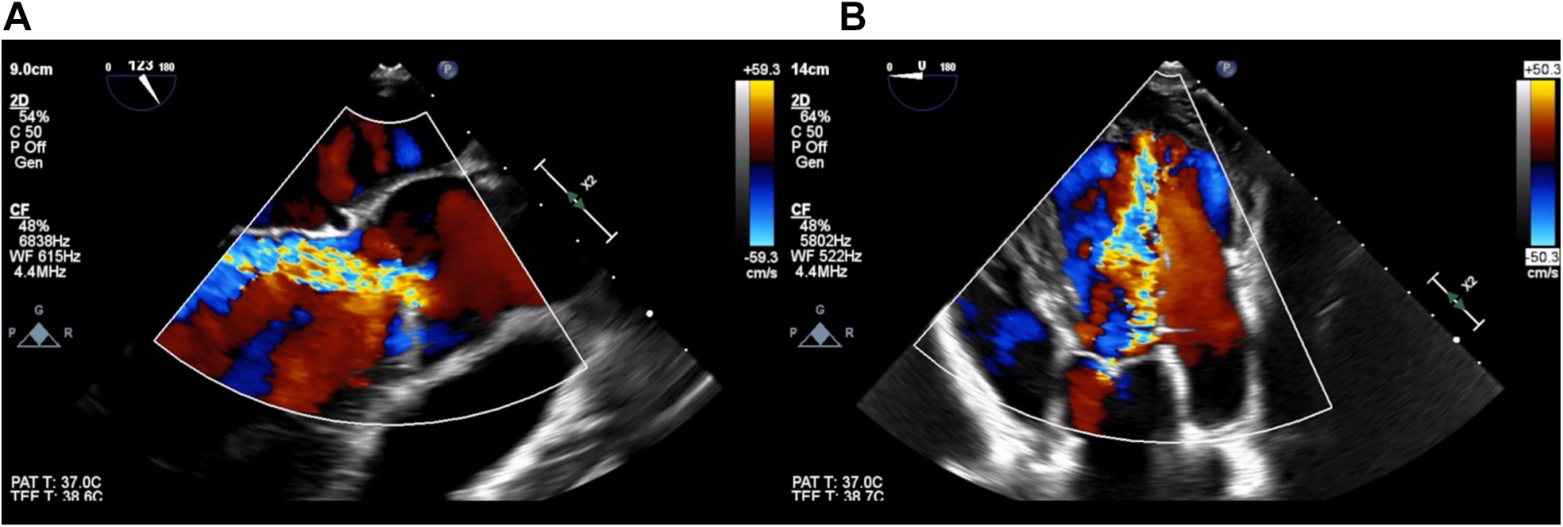
Transesophageal Echocardiography (A) Midesophageal long-axis view and (B) transgastric view showing severe aortic regurgitation. CF = color flow; WF = frequency sound waves; other abbreviations as in Figure 1.

**Figure 3:**
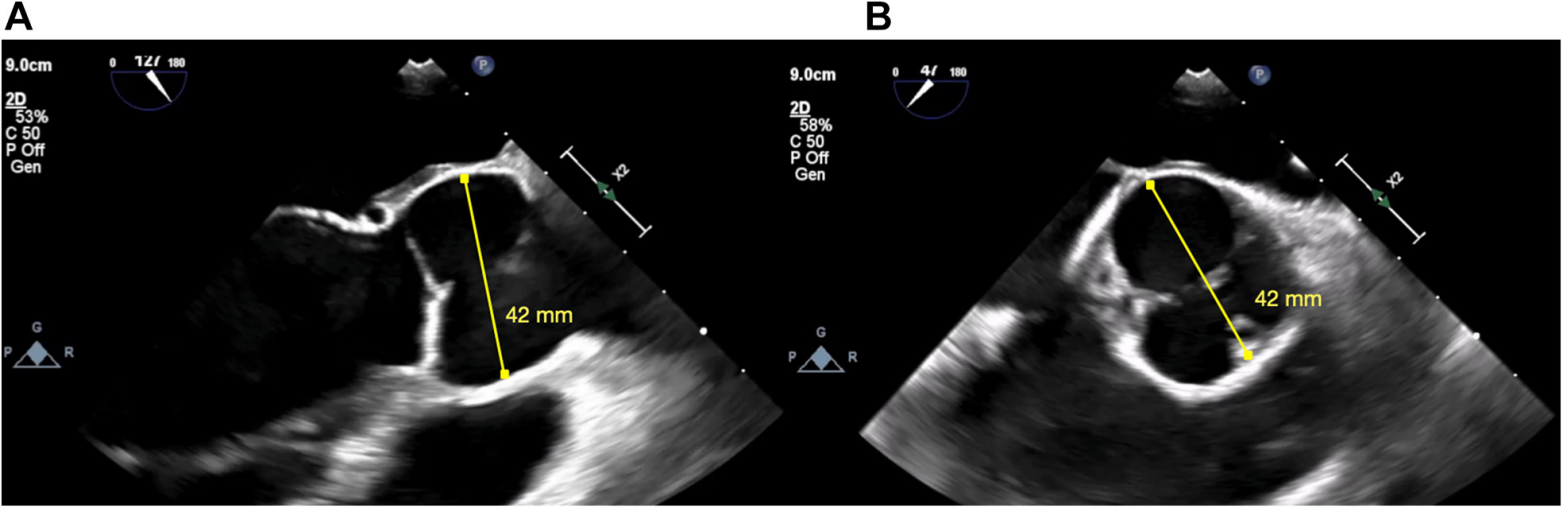
Aortic Root Diameter on Transesophageal Echocardiography (A) Midesophageal long-axis view and (B) midesophageal aortic valve short-axis view with a maximum aortic root diameter of 42 mm. Abbreviations as in Figure 1.

**Figure 4:**
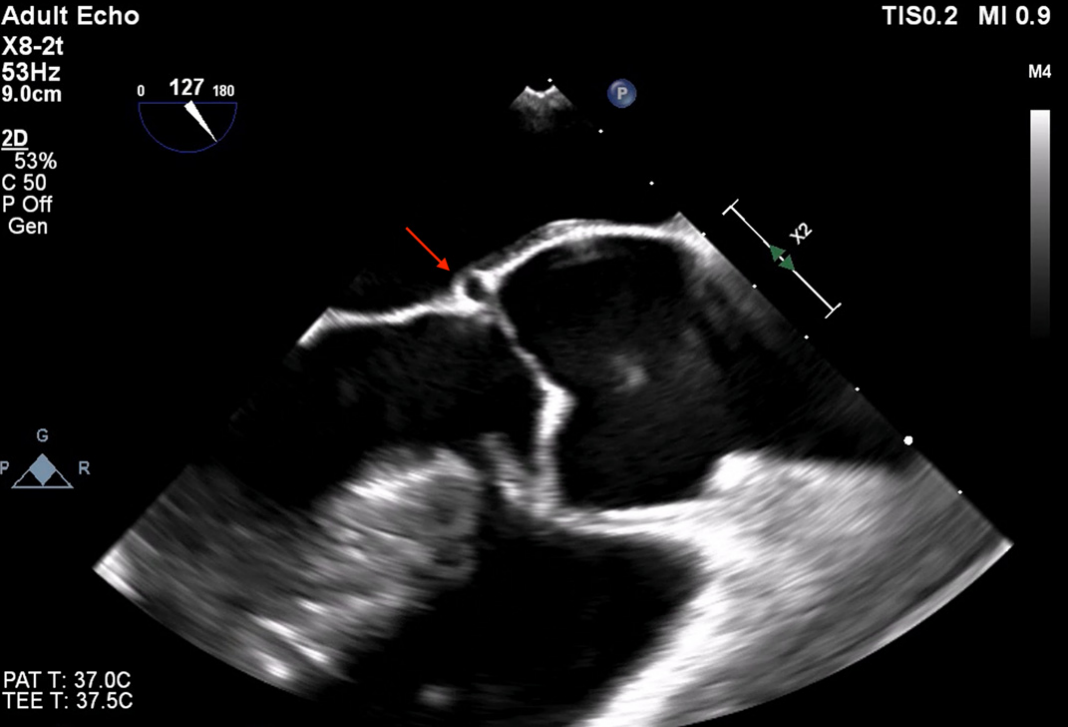
Midesophageal Long-Axis View The aberrant circumflex coronary artery at the level of the aortic annulus is visible (arrow). Echo = echocardiogram; other abbreviations as in Figure 1.

**Figure 5:**
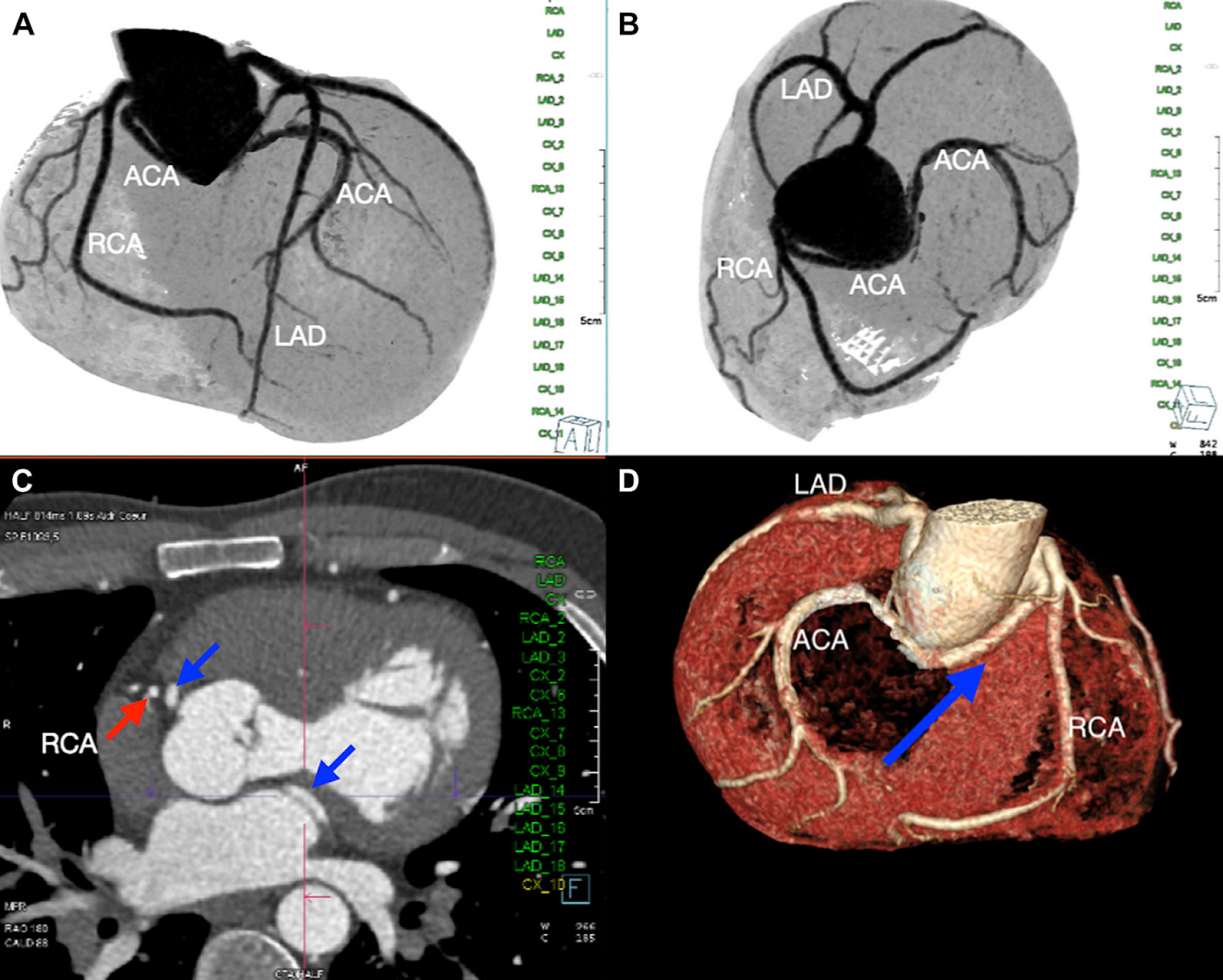
Computed Tomography of the ACA Computed tomography showing the pathway of the ACA (A, B) originating from the right coronary sinus and passing laterally along the non-coronary sinus. (C) The origin of the ACA is alongside the ostium of the right coronary artery (red arrow). (D) 3 D reconstruction of the pathway of the aberrant circumflex artery (blue arrow). ACA = aberrant circumflex artery (blue arrow) arising from the right coronary ostium; LAD = left anterior descending coronary artery; RCA = right coronary artery (red arrows).

Transthoracic echocardiography showed a severely dilated left ventricle (end-diastolic diameter, 69 mm; end-systolic diameter, 47 mm) with mildly depressed left ventricular (LV) function (ejection fraction [EF]: 50%). Moreover, the aortic valve phenotype was a De Kerchove type A BAV^1^ (Figure 1), with left-right cusp fusion and a typical fused cusp prolapse with a posteriorly directed eccentric jet (regurgitant volume, 63 mL; EROA, 30 mm^2^) (Figures 2A and 2B). The root was dilated at 42 mm (Figures 3A and 3B, Video 1). The transesophageal echocardiogram indicated an anomalous path of a coronary artery (Figure 4), thus prompting a computed tomography (CT) angiogram. This imaging identified the presence of an aberrant circumflex artery (ACA), originating from the right coronary sinus and passing laterally along the noncoronary sinus and behind the aortic root (Figure 5).

A multidisciplinary heart team discussion determined the patient to be a suitable candidate for a valve-sparing root replacement (VSRR) despite the anomalous coronary artery.

## Surgical Management

After a full sternotomy and initiation of cardiopulmonary bypass, the aorta was cross-clamped, and antegrade intermittent warm blood cardioplegia was delivered directly into the 3 ostia (Video 1). In fact, at the level of the proximal right coronary artery we found a short main trunk, and the circumflex artery originated at the side of the right coronary artery with a separate ostium. Valve analysis confirmed a BAV with a raphe between the left and right leaflets and a symmetrical phenotype.

**Figure 6:**
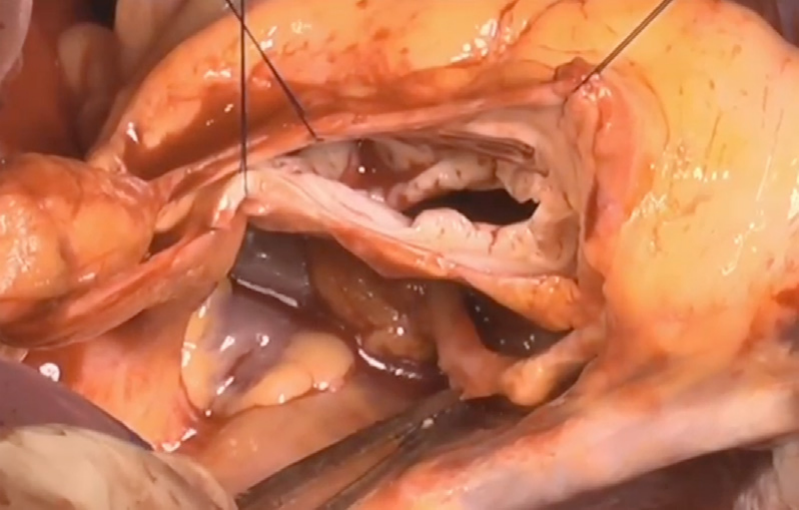
Aberrant Circumflex Artery Mobilized Mobilization and dissection of the aberrant circumflex artery along its entire course before the reimplantation of the valve.

The aortic root was carefully dissected,^2^ and the ACA was dissected free in its entire proximal course. It was thus mobilized off the aortic root: from its origin, in close proximity to the commissure between the right coronary cusp and the noncoronary cusp, to the posterior aspect of the noncoronary and left coronary sinus, overlying the left atrial dome, to allow for safe suture placement later (Figure 6). We ended the dissection at the level of roof of the left atrium, where the artery passes into the sinus oblique and takes its natural course for perfusion of the lateral wall.

The aortic repair performed was a reimplantation technique specifically for BAVs with a 28-mm Valsalva Cardioroot prosthesis (Getinge), which has previously been described in detail.^2^^,^^3^ The fused cusp prolapse was treated with a central plication suture of the fused cusp.

**Figure 7:**
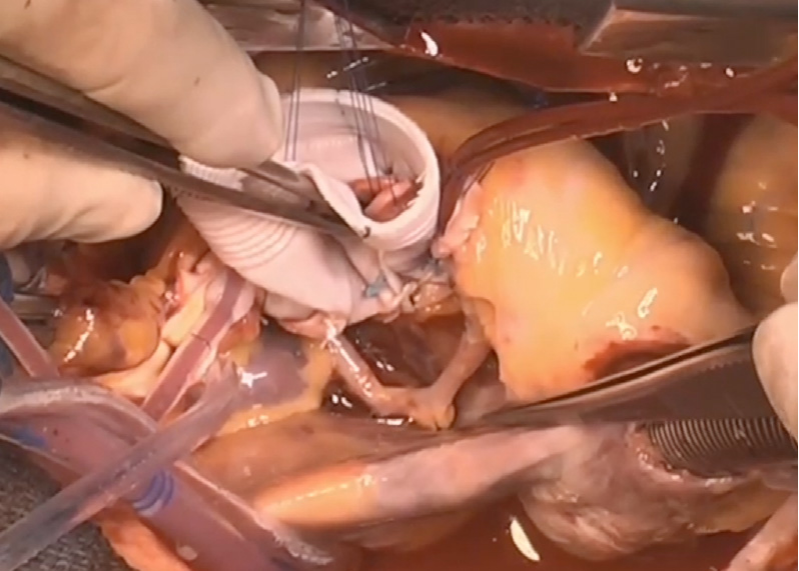
Aberrant Circumflex Artery Mobilized After Valve-Sparing Root Replacement Mobilization and dissection of the aberrant circumflex artery along its entire course at the end of the reimplantation of the valve into the Valsalva graft.

Following completion of valve repair and reimplantation, we verified that the circumflex artery was free and did not become kinked during suture placement (Figure 7).

The left coronary button was then reimplanted in the anatomic position in the left neo-sinus of the graft. The right coronary button with the right and circumflex ostia was then reimplanted into the neo-right sinus (Video 2).

At the end of the procedure, intraoperative echocardiography showed a symmetrical BAV with no regurgitation and good cusp mobility with a peak gradient of 10 mm Hg (Video 3) and preserved lateral wall motion.

The patient was extubated on day 0, had an uneventful postoperative course, and was discharged home on postoperative day 5.

## Follow-Up

Follow-up echocardiography at 1 month showed no AR with a peak transvalvular gradient of 7 mm Hg, no lateral wall motion abnormalities, and improvement in LV function (LV ejection fraction [LVEF], 45%-50%) (Video 4).

## Discussion

BAVs represent the most common congenital cardiac malformations,^4^ often manifesting with AR, and can be associated with coronary anomalies such as higher ostial locations, a short left main trunk, or left coronary arterial dominance.^5^

This particular coronary anomaly in itself is typically asymptomatic. Symptoms and the risk of sudden death are more prevalent when the origin of a right coronary artery arises from the left sinus with an associated intramural and/or interarterial course.

The location of an ACA runs typically at the level of the noncoronary sinus annulus with posterior orientation and thus within the dissection plane for a VSRR, a location that renders an abnormal coronary artery at high risk for injury during aortic surgery.^6^

In the current literature there are very few reports of concomitant BAVs and ACAs.^6^, ^7^, ^8^ To our knowledge, this is the first case of a surgical repair of this entity with VSRR and valve repair. Preoperative work-up including cardiac CT angiography helps to identify coronary anomalies and anticipate potential intraoperative risks. Abnormal coronary arteries either can be reimplanted in a more physiologic location, when performing an aortic root replacement, or can be ligated and bypassed if needed. Reimplantation into a more anatomically correct position, however, can be associated with kinking of the reimplanted artery, as reported by Urbanski et al,^9^ and should therefore be approached with care. Furthermore, the ACA originates from a common ostium with the right coronary artery, and thus it cannot be reimplanted without compromising 1 of the 2 vessels.^7^ Unroofing is no option here either because there is no intramural course of the aberrant vessel.

According to the 2020 American College of Cardiology/American Heart Association guidelines for valvular heart disease, in asymptomatic patients with severe AR and depressed LV systolic function (LVEF ≤55%) valvular surgery is indicated (Class 1, Level of Evidence: B); and in patients with BAV, repair can be considered if it is performed at a comprehensive valve center such as ours (Class 2, Level of Evidence: B-C). However, there are no guideline-directed recommendations for BAV and associated coronary anomalies because these occur only rarely.^9^

Skeletonization and full mobilization of the vessel from its origin along its entire course up to the atrioventricular groove are key to reducing the risk of coronary injury related to suture placement or vessel compression related to the prosthetic graft.^10^

Baldonado et al^6^ reported a 16% risk of myocardial ischemia on echocardiography after aortic valve replacement in patients with ACA. The successful management of this case highlights the notion that even in the presence of an ACA with a severely regurgitant BAV, VSRR and valve repair are feasible.

Our study received the proper ethical oversight.

## Conclusions

VSRR can be safely performed in patients with an ACA. CT angiography or coronary artery angiography is important for surgical planning, even in young patients.

## Funding Support and Author Disclosures

The authors have reported that they have no relationships relevant to the contents of this paper to disclose.

## Appendix

Video 1 Preoperative Transthoracic Echocardiogram Showing Severe AR and Aortic Root Dilatation

Video 2a Surgical Procedure Valve-sparing root replacement of the bicuspid valve in a Cardioroot 28-mm prosthesis (Getinge) with the skeletonization and mobilization of the aberrant circumflex artery

Video 2b Surgical Procedure Valve-sparing root replacement of the bicuspid valve in a Cardioroot 28-mm prosthesis (Getinge) with the skeletonization and mobilization of the aberrant circumflex artery

Video 2c Surgical Procedure Valve-sparing root replacement of the bicuspid valve in a Cardioroot 28-mm prosthesis (Getinge) with the skeletonization and mobilization of the aberrant circumflex artery

Video 2d Surgical Procedure Valve-sparing root replacement of the bicuspid valve in a Cardioroot 28-mm prosthesis (Getinge) with the skeletonization and mobilization of the aberrant circumflex artery

Video 3 Postoperative Transesophageal Echocardiogram Highlighting a Perfect Repair Result, With No AR and Mildly Depressed Left Ventricular Function

Video 4 Follow-Up Echocardiography Demonstrating the Good Movement of the Aortic Leaflets With No AR and Good Function of the Left Ventricle
